# Ablative fractional carbon dioxide laser combined with intense pulsed light for the treatment of photoaging skin in Chinese population: A split-face study

**DOI:** 10.1097/MD.0000000000009494

**Published:** 2018-01-19

**Authors:** Xue-Ling Mei, Li Wang

**Affiliations:** Department of Dermatology, Beijing Friendship Hospital, Capital Medical University, Beijing, China.

**Keywords:** Asian skin, fractional laser, intense pulsed light, laser resurfacing, photoaging, photorejuvenation, split-face

## Abstract

Intense pulsed light (IPL) is effective for the treatment of lentigines, telangiectasia, and generalized erythema, but is less effective in the removal of skin wrinkles. Fractional laser is effective on skin wrinkles and textural irregularities, but can induce postinflammatory hyperpigmentation (PIH), especially in Asians. This study evaluated the safety and efficacy of ablative fractional laser (AFL) in combination with IPL in the treatment of photoaging skin in Asians.

This study included 28 Chinese women with Fitzpatrick skin type III and IV. The side of the face to be treated with IPL alone (3 times) or AFL in combination with IPL (2 IPL treatments and 1 AFL treatment) was randomly selected. Skin conditions including hydration, transepidermal water loss, elasticity, spots, ultraviolet spots, brown spots, wrinkle, texture, pore size and red areas, as well as adverse effects were evaluated before the treatment and at 30 days after the treatment.

Compared with IPL treatment alone, AFL in combination with IPL significantly increased elasticity, decreased pore size, reduced skin wrinkles, and improved skin texture (*P* = .004, *P* = .039, *P* = .015, and *P* = .035, respectively). Both treatment protocols produced similar effects in relation to the improvement of photoaging-induced pigmentation. The combined therapy did not impair epidermal barrier function. No postoperative infection, hypopigmentation, or scarring occurred after IPL and AFL treatments. PIH occurred at 1 month after AFL treatment and disappeared at 30 days after completion of the combined therapy.

AFL in combination with IPL is safe and effective for photoaging skin in Asians.

## 1 Introduction

Skin aging is a degenerative process that results in wrinkles, lentigines, keratoses, dyspigmentation, telangiectasia, decreased elasticity, textural roughness, and sallow color.^[1]^ The pathology of skin aging is complex, and both intrinsic and extrinsic factors contribute to the pathogenesis of skin aging. Intrinsic (chronological) aging is caused by inherited genes, and extrinsic factors (also called photoaging) are caused by many external factors such as sun exposure.^[2]^ With an increase in the aging population, more people are seeking to maintain skin health and the demand for aging skin treatment has increased.

Intense pulsed light (IPL) therapy has been reported to be effective in the treatment of aging skin.^[3–6]^ IPL therapy is a low-energy, low-density, nonablative therapy using continuous intense pulsed photons. IPL uses noncoherent broad spectrum light generated from high-powered Xenon lamps. Using filters, the associated spectrum can be narrowed, thereby optimizing the therapeutic parameters such as wavelength, pulse duration, and energy for specific tissues. IPL penetrates the tissues, and is selectively absorbed by melanin and hemoglobin, thereby producing photothermal effects. IPL can cause reversible thermal damage to collagen, and induce contraction of collagen fibers and fiber remodeling; this phenomenon results in photorejuvenation effects. It has been reported that IPL treatment is effective for the treatment of facial pigmentation and vascular lesions.^[2,7]^

Conventional carbon dioxide (CO_2_) and erbium: yttriumaluminum-garnet lasers, which can remove the stratum corneum, has been regarded as the gold standard for skin laser resurfacing.^[8]^ However, the clinical application of these lasers is restricted due to many side effects such as damage to the entire epidermis, postoperative infection, dyspigmentation, prolonged recovery period, and scar formation.^[9]^ Fractional lasers (FL), including ablative fractional lasers (AFL) and nonablative fractional lasers (NAFL), have been widely used in cosmetic practices,^[10,11]^ and are effective for the treatment of aging skin.^[5]^ The concept of fractional photothermolysis (FP) was first introduced in 2004, and the light emitted from the FP device produces multiple microscopic treatment zones (MTZ) and induces thermal injury to the skin.^[12]^ The self-repair of the damaged MTZ promotes collagen regeneration, which results in an improvement in skin texture. In addition, the small zones of injury can be easily repaired by peripheral normal skin tissues. It has been reported that during the process of wound healing, AFL-induced microscopic epidermal necrotic debris (MEND) promote epidermal heat-shock protein synthesis and collagen remodeling, which can last for more than 3 months.^[13]^ Furthermore, AFL has been found to induce the upregulation of metalloproteinases-1 and 3 and procollagens I and III at 24 h after treatment. This upregulation plays an important role in wound-healing. Keratinocyte migration can result in re-epithelialization within 24 h after AFL, and MEND and epidermal barrier function can be recovered within 7 days after AFL.^[10,14]^ Therefore, FL is effective in the treatment of photoaging skins with minimal risks and short recovery time.^[15,16]^

IPL is widely used for photorejuvenation and is effective in the treatment of skin diseases such as lentigines, telangiectasia, and generalized erythema, but is less effective in the removal of actinic keratoses, flat seborrheic keratoses, and skin wrinkles.^[4,17–19]^ By contrast, FL produces good therapeutic effects on skin wrinkles, textural irregularity, and early actinic keratoses. However, FL can induce postinflammatory hyperpigmentation (PIH), especially in Asians.^[11,20]^ FL combined with IPL therapy is expected to produce better efficacy with less side-effects for the treatment of photoaging skin. Kearney and Brew^[3]^ reported that NAFL in combination with IPL produced a satisfactory clinical outcome with a good safety profile. However, the efficacy and safety of AFL in combination with IPL in the treatment of photoaging skin is rarely reported in the literature.^[9,21]^ In this study, we aimed to investigate the efficacy of AFL in combination of IPL in the treatment of photoaging skin in Chinese people. We systematically evaluated the effect of the combined therapy in relation to multiple parameters including skin elasticity, pigmentation, wrinkle, texture, pore size, red areas, hydration, and transepidermal water loss (TEWL). The purpose of this study was to establish a safe and effective combined therapy for the treatment of photoaging skin in Asians.

## 2 Materials and methods

### 2.1 Subjects

The Institutional Review Board of Beijing Friendship Hospital affiliated to the Capital Medical University approved this study, and all subjects gave their informed consent prior to their inclusion in the study. The eligible subjects in the study were women aged between 40 and 60 years; the recruited females had Fitzpatrick skin types III and IV and were in generally good health. The exclusion criteria were as follows: recent exposure to the sun; patients with light sensitivity or porphyria who were unable to undergo light therapy; progressive psoriasis, vitiligo, or keloids and hypertrophic scars; autoimmune diseases or serious systemic diseases; malignant tumors or precancerous lesions in the skin or other organs; damage or infection in the skin for light therapy; herpes simplex infection in the previous 6 months; the use of photosensitive drugs or topical retinoids in the previous 4 weeks; the use of systemic corticosteroids or oral retinoids in the previous 6 months; facial laser photon beauty or similar treatments in the previous 6 months; facial filling or wire implantation; the use of oral contraceptives; and pregnancy and lactation.

### 2.2 Treatment protocol

The participants received IPL alone in one side of the face or in combination with AFL in the other side of the face. The side of the face to be treated with IPL alone or in combination with AFL was randomly selected in accordance with a computer-generated randomization schedule. The side of the face treated with ILP alone received IPL treatment 3 times on days 0, 30, and 90. The side of the face treated with IPL and AFL received IPL on day 0, followed by AFL on day 30, and IPL on day 90. The participants were followed up at 30 days after complete therapy.

### 2.3 IPL procedure

Chilled colorless gel (1–2 mm thick) was evenly applied to the treated area to avoid the IPL-induced damage on the epidermis and dermis. Based on the Fitzpatrick skin type, the skin was irradiated with an IPL source (Lumenis One, Lumenis Ltd., Yokneam, Israel) with a 590 or 640 nm filter (range, 515–1200 nm). The parameters used were as follows: double pulse mode; pulse durations, 4.0 to 4.5 ms; pulse delays, 30 to 35 ms; and energy density, 14 to 17 J/cm^2^. The efficacy of the treatment was evaluated immediately after the treatment, and the treatment was considered to be effective if redness of the skin occurred and the patients experienced a mild burning sensation. After the treatment, the skin was covered with ice for 30 min, and the side effects were observed. The participants were asked to avoid direct exposure to sunlight.

### 2.4 AFL procedure

The face was anesthetized with topical anesthetic cream (2.5% lidocaine and 2.5% prilocaine; Tsinghua Ziguang Co., Beijing, China) at 1 h before the AFL. The skin was irradiated with a fractional carbon dioxide laser (Pixel CO_2_, Alma Lasers Ltd., Israel). The laser parameters were as follows: single pass; pulse width, 1.2 ms; spot size, 250 μm; fluence, 84 mJ/pixel, and treatment density, 10%. After the treatment, the skin was covered with ice for 30 min twice daily until the edema disappeared.

### 2.5 Follow-up and clinical evaluation

All subjects were evaluated before the treatment (baseline) and at 30 days after the treatment. All patients cleaned their faces and rested for 20 min in a quiet room at room temperature (22–24°C) and a humidity of 40% to 50% before evaluation. TEWL, skin hydration, and elasticity were measured on the surface of both cheeks using the Cutometer dual MPA580 (Courage + Khazaka, Cologne, Germany).

Photographs were systematically taken, using VISIA System (VISIA Facial Imaging Device, Canfield Scientific Inc.) under different illumination conditions including Standard flash, Cross Polarised, and Ultra violet-A lights. Images taken under illumination with the standard light source were used to analyze the wrinkles, spots, texture, and pore size of the skin. Images taken under illumination with the UVA light source were used to analyze the ultraviolet spots to determine the melanin formation in the epidermis. Images taken under illumination with a cross-polarized light source were used to analyze brown spots and red areas to determine the formation of melanin and hemoglobin in the deep layer of the skin.^[22]^ The feature count values were recorded for comparison.

Adverse reactions were examined before the treatment (baseline) and at 30 days after the treatment.

### 2.6 Statistical analysis

Statistical analyses were performed using SPSS 17.0 (SPSS, Inc., Chicago, IL). All values are presented as mean ± standard deviation. A paired *t* test was used to compare the difference in the parameters before and after treatment in the same treatment group. In comparisons between groups, parametric tests and independent samples *t* tests were used for analysis, the level of statistical significance was set at 0.05.

## 3 Results

Thirty female volunteers with photoaging skin were initially enrolled in this study, and 28 subjects completed the study. Two subjects were lost to follow-up and were excluded from the study. The average age of the 28 subjects with Fitzpatrick skin types III and IV was 51.4 years (range, 41–60 years).

**Figure 1:**
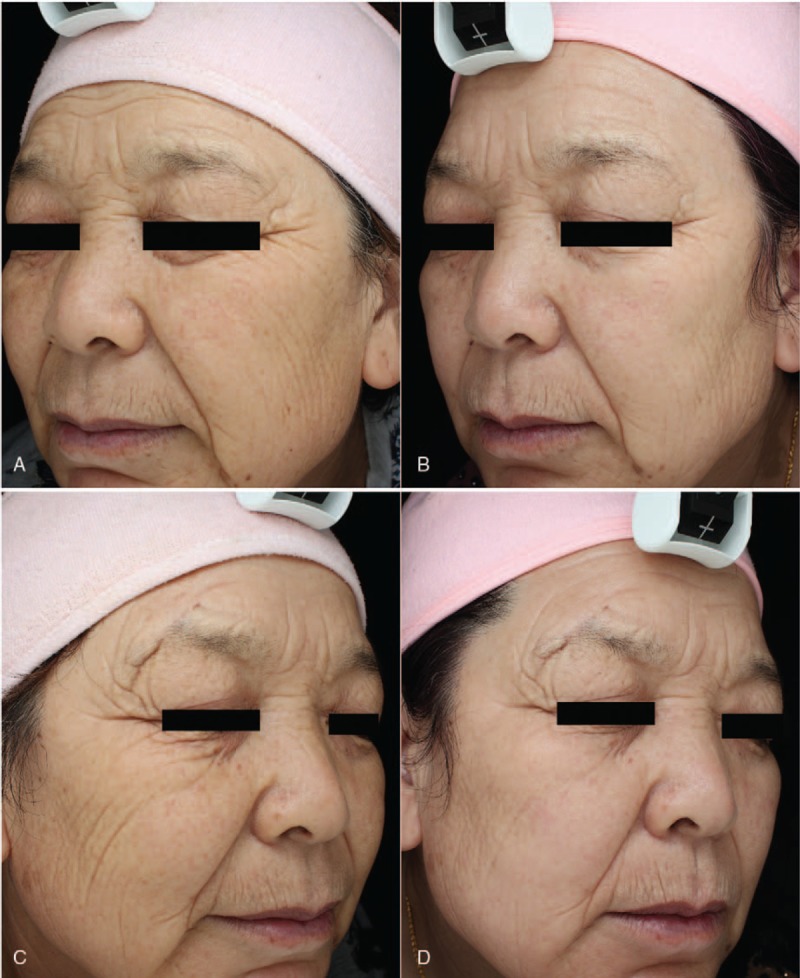
Photographic evidence before (A, C) and after (B, D) IPL alone (A, B) on the left face and AFL in combination with IPL on the right face (C, D) from patient 1.

**Figure 2:**
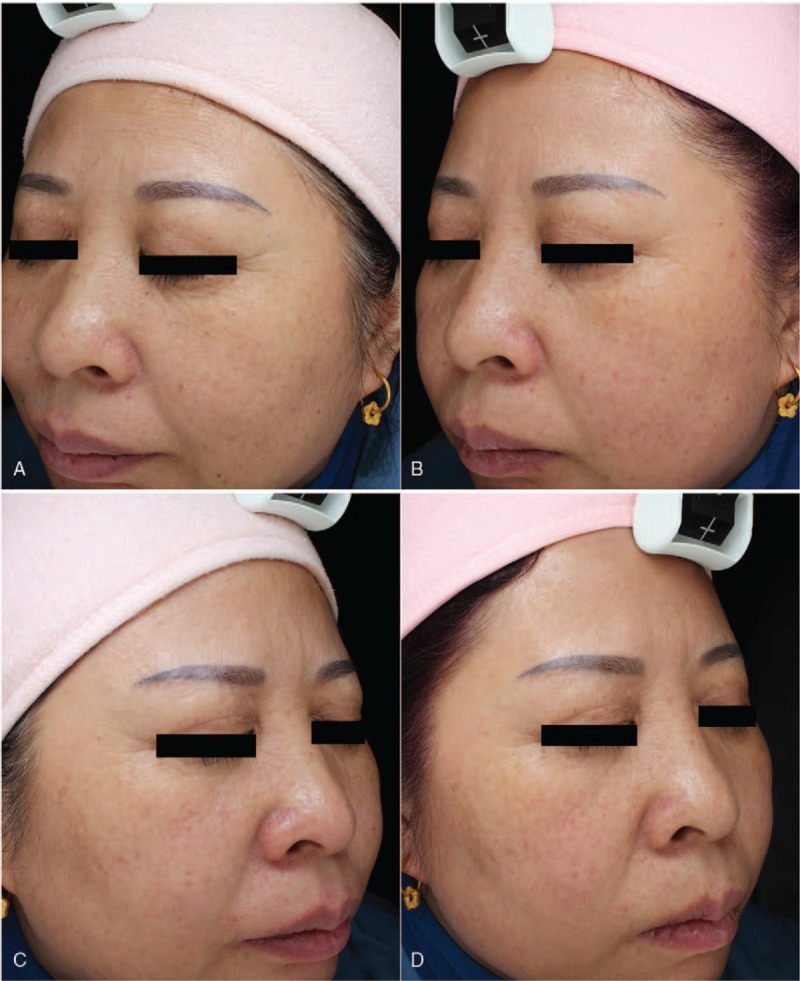
Photographic evidence before (A, C) and after (B, D) AFL in combination with IPL (A, B) on the left face and IPL alone on the right face (C, D) from patient 2.

Table 1 summarizes the effect of IPL alone and AFL in combination with IPL in the treatment of photoaging skin. The skin conditions including hydration, TEWL, elasticity, pigmentation, wrinkle, texture, pore size, and red areas were analyzed and compared before and after IPL treatment alone or AFL in combination with IPL. Compared with before treatment, IPL treatment alone, and AFL in combination with IPL significantly increased elasticity, reduced pore size, decreased red areas, and improved pigmentation-associated parameters. In addition, compared with before treatment, AFL treatment in combination with IPL, but not IPL treatment alone, significantly reduced skin wrinkle and improved skin texture (Table 1 and Figs. 1 and 2).

**Table 1:**
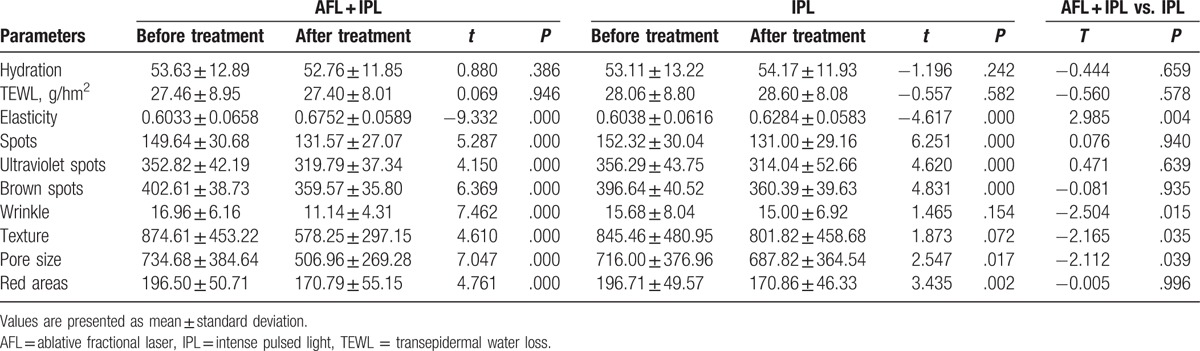
Evaluation of the effect of IPL alone and AFL in combination with IPL in the treatment of photoaging skin.

We then compared the differences between IPL treatment alone and IPL treatment combined with AFL for the treatment of photoaging skin. There were no significant differences in pigmentation, elasticity, wrinkle, texture, pore size, and red areas before the treatment between IPL treatment and AFL treatment groups. Compared with IPL treatment alone, AFL treatment in combination with IPL significantly increased skin elasticity, decreased pore size, reduced skin wrinkles, and improved skin texture. There were no significant differences in skin hydration, TEWL, red areas, and pigmentation-associated parameters between the 2 groups (Table 1 and Figs. 1 and 2).

The values of skin hydration and TEWL before the treatment were not significantly different from those after treatment in the IPL treatment and AFL treatment groups. There were no significant differences in skin hydration and TEWL before and after the treatment between IPL treatment and AFL treatment groups.

All participants had no severe and persistent adverse effects. Mild burning sensation, pain and transient erythema occurred in the skin treated with IPL alone and relieved with ice pack for 30 min. All skins had spotted epidermal exfoliation and edema immediately after AFL treatment and scar tissues were formed 1 day after AFL treatment. Edema, oozing, and crusting spontaneously disappeared at 3 to 7 days after AFL treatment, and erythema lasted for 7 to 14 days. PIH occurred at 1 month after AFL treatment in 9 (32%) cases. PIH completely disappeared at 30 days after the treatment in 8 cases, and the persistent presence of PIH occurred in 1 case. No blisters, postoperative infection, hypopigmentation, or scarring occurred during and after IPL and AFL treatments.

## 4 Discussion

In this study, we compared the effectiveness and safety of AFL therapy in combination with IPL and IPL therapy alone in the treatment of photoaging skin in 28 Chinese women. We observed that compared with IPL treatment alone, AFL treatment in combination with IPL significantly increased skin elasticity, decreased pore size, reduced skin wrinkle and improved skin texture. Conversely, both treatments produced similar effects in relation to skin hydration, TEWL, red areas, and pigmentation. In addition, the combined therapy was not associated with severe adverse reactions. Our findings suggest that AFL combined with IPL is a safe and effective treatment for photoaging skin in Asians.

As a mechanical barrier, the skin can prevent the loss of water, electrolytes, and proteins from the body. The barrier dysfunction of the skin is mainly manifested by increased TEWL and decreased hydration of the skin.^[23,24]^ It has been reported that TEWL was significantly increased at 3 days after AFL treatment and recovery to the normal level at 4 weeks after therapy.^[25]^ In this study, we found that skin hydration and TEWL at 1 month after AFL combined with IPL was similar to those before the treatment, and was not significantly different from those after IPL treatment alone, suggesting that AFL in combination with IPL does not significantly impair skin barrier function.

Compared with Caucasians, Asians with Fitzpatrick skin types III and IV have higher melanin content, thus rendering Asians susceptible for PIH after FL treatment. Therefore, the optimal treatment strategy for photoaging skin in Asians is to ensure the effectiveness of the treatment with a low risk for PIH.^[26]^ It has been reported that IPL is effective at reducing pigmentation in the skin.^[27,28]^ Consistent with these reports, we found that IPL treatment alone significantly improved pigmentation-associated parameters including skin spots, ultraviolet spots, and brown spots. In addition, AFL treatment in combination with IPL produced similar effects on reducing pigmentation spots. In the combined therapy, IPL treatment was performed 2 months after AFL treatment. The purpose of IPL treatment was to promote PIH regression. Of the 28 cases, PIH occurred at 1 month after AFL treatment in 9 (32%) cases, and no PIH occurred in faces treated with IPL alone. At the last follow-up, PIH completely disappeared in 8 cases, and only 1 case had persistent PIH after the combined therapy. In accordance with our results, Kearney and Brew reported that NAFL in combination with IPL produced satisfactory outcomes with no persistent PIH in the treatment of photoaging skin in the Australian population.^[3]^ Our results suggest that AFL in combination with IPL can promote PIH regression, and is not associated with the persistent presence of PIH in Asians.

The occurrence of PIH is associated with a high treatment density of AFL, and cooling treatment can effectively reduce PIH occurrence by preventing bulk tissue heating.^[29]^ In this study, we adopted a low treatment density of AFL (10%) and the treatment area was covered with ice for 30 min twice daily until the edema disappeared. Despite these strategies, the incidence of PIH at 1 month after AFL treatment was still as high as 32%, which is higher than that (8.3–25%) reported in the literature.^[30–32]^ Although PIH disappeared at the last follow-up in most cases after IPL treatment, and there was no significant difference in pigmentation-associated parameters (skin spots, ultraviolet spots, and brown spots) between faces treated with IPL alone and AFL in combination with IPL, the occurrence of transiently high incidences of PIH after AFL treatment suggest that therapy parameters including treatment density, spot energy, pulse width, and spot size need to be optimally adjusted.

The efficacy and safety of FL combined with other laser for the treatment of photoaging skin in Asians are rarely reported in the literature.^[9,21]^ AFL has been found to be superior to NAFL in reducing photoaging-induced wrinkles, and the former approach effectively reduces textural irregularity in photoaging skins.^[33–35]^ The skin tightening effect of AFL therapy may be due to its penetration into the deep layer of the dermis, where it stimulates fibroblast proliferation and promotes rearrangement of collagen and elastin.^[31]^ Skin ultrasonography demonstrates that the thickness and density of the dermis is increased after AFL treatment, but not after NAFL treatment.^[25]^ To date, AFL has been regarded as the optimal treatment option for photoaging skin by many dermatologists.^[36,37]^ AFL can produce mild to moderate effects on reducing facial skin wrinkles after 1 to 2 treatments, whereas NAFL produces similar effects after 5 to 6 treatments.^[11]^ In this study, the combined therapy including a single AFL treatment and 2 IPL treatments, significantly reduced skin wrinkles and improved skin texture, whereas 3 IPL treatments did not significantly improve skin wrinkles and texture. In addition, the combined therapy significantly increased elasticity and decreased pore size compared with IPL treatment alone. Our study suggests that AFL treatment in combination with IPL is superior to IPL treatment alone in the treatment of photoaging skin.

Compared with NAFL, although AFL produces better clinical outcomes, it can induce greater skin damage, and is associated with a higher risk of postoperative adverse effects such as prolonged erythema, infection, hypopigmentation, hyperpigmentation, and scarring.^[29]^ Aggressive AFLs (fluence, 52.5–200 mJ, treatment density, >30–40%) are associated with a higher risk of these adverse effects.^[31,32,38]^ In this study, we used less aggressive AFL (fluence, 84 mJ/pixel, and treatment density, 10%) in combination with IPL and found that the combined therapy was not associated with severe adverse effects such as infection, hypopigmentation, and scarring in all 28 cases. Erythema lasted for 7 to 14 days after the combined therapy and disappeared spontaneously.

This study has some limitations. First, the sample size of this study (n = 28) is relatively small. Therefore, future studies with a large sample size should be performed to confirm the conclusion of this study. Second, we found that the incidence of PIH at 1 month after AFL treatment was higher than that reported in the literature.^[30–32]^ This high incidence may be associated with the small sample size utilized in this study, which may have resulted in a biased result. Third, the patients were only followed up for 1 month. The long-term efficacy and safety of AFL treatment in combination with IPL for photoaging has not been investigated. Further studies will be performed to investigate the long-term effect of the combined therapy for the treatment of photoaging skin in Asians.

## 5 Conclusion

In summary, we found that AFL treatment in combination with IPL significantly reduced skin wrinkles and pore size and improved skin texture and elasticity in photoaging skin of Chinese people. In addition, the combined therapy was also effective in treating photoaging-induced hyperpigmentation, telangiectasia, and generalized erythema. Furthermore, the combined therapy did not impair skin barrier function. Although PIH occurred after AFL treatment, it disappeared at 30 days after combined therapy in most cases. No severe adverse effects such as infection, hypopigmentation, and scarring occurred after the combined therapy. Therefore, AFL combined with IPL is a safe and effective treatment for photoaging skin in Asians.
